# Metagenomic and functional insights into root endophytic bacteria associated with drought stress in cowpea

**DOI:** 10.1038/s41598-026-45459-4

**Published:** 2026-03-22

**Authors:** Boshra Ahmed Halo, Yaqeen A. S. Aljabri, Bernard R. Glick, Mahmoud W. Yaish

**Affiliations:** 1 Department of Biology, College of Sciences, Sultan Qaboos University, Alkhoud, P.O. Box 36, 123 Muscat, Oman; 2 Department of Biology, University of Waterloo, Waterloo, ON N2L 3G1 Canada

**Keywords:** Amplicon sequence variants, Cowpea, Cyanobacteria, Drought, Endophytic bacteria, Microbiome, Sustainability, Plant sciences, Microbial ecology, Microbiome

## Abstract

Endophytic bacterial communities enhance plant drought resilience, yet their dynamics in cowpeas (*Vigna unguiculata*) remain poorly understood. To explore this, we analyzed the root endophytic bacteriome under drought stress using 16 S rRNA gene metagenomics and evaluated isolated bacteria for plant growth-promoting traits. Drought significantly reduced both alpha and beta diversity, indicating a loss of microbial richness and evenness and community homogenization. Taxonomic shifts revealed enrichment of Cyanobacteriota, Cyanophyceae, and *Marileptolyngbya sina* in drought conditions. Forty-seven endophytic isolates were identified and characterized, including *Enterobacter* spp., *Bacillus* spp., *Leclercia adecarboxylata*, and *Stenotrophomonas* spp. The isolated strains exhibited plant growth-promoting traits in vitro and, in a pot assay, some enhanced wheat biomass under both control and drought conditions. The reduction in diversity due to drought indicates a loss of microbial richness and evenness, along with homogenization of microbial composition, suggesting that drought selectively enriches specific taxa, which may enhance plant stress resilience through specialized metabolic functions. By combining metagenomic profiling with functional assays, this study highlights the role of drought-induced bacterial shifts in supporting plant growth and development. These insights could lead to the development of microbial inoculants to improve crop drought tolerance.

**Supplementary Information:**

The online version contains supplementary material available at 10.1038/s41598-026-45459-4.

## Introduction

Drought is one of the most widespread environmental stresses, posing a significant challenge to global agriculture by severely limiting plant growth and productivity^1,2^. In regions like the Middle East, rising temperatures and declining precipitation have intensified the impacts of drought, further threatening agricultural sustainability, even for drought-tolerant crops such as cowpeas^3–6^.

Cowpea (*Vigna unguiculata* L. Walp.), a drought-tolerant legume widely cultivated in arid and semiarid regions, has a significant agronomic and nutritional value. This plant is rich in nutrition, used as a staple crop, and a vital protein source in many developing countries^7^. Its ability to grow under various abiotic stress conditions makes it an essential component of sustainable cropping systems, especially in drought-prone zones^8,9^. However, cowpea drought resilience varies across genotypes, with some exhibiting substantial yield losses under water-limited conditions^10^. For example, drought was reported to reduce cowpea grain and fodder yields by approximately 62% and 56%, respectively^11^. In fact, the increasing frequency and severity of droughts, intensified by climate change, are causing more substantial crop losses. Consequently, there is an urgent need for innovative strategies to enhance cowpea productivity under these adverse conditions, including the use of biostimulants such as growth-promoting microorganisms.

Endophytic bacteria are non-pathogenic microbes that reside within plant tissues and play a vital role in enhancing plant growth and stress tolerance^12^. Unlike epiphytic bacteria, which colonize external surfaces, endophytic bacteria interact directly with plant metabolism, providing multifaceted benefits such as enhanced nutrient acquisition, phytohormone production, and improved water-use efficiency^13^. These microbes hold particular promise for addressing drought stress, as they can enhance root system architecture, synthesize osmoprotectants, and modulate stress-responsive gene expression, thus enabling plants to cope with water scarcity^14,15^, in collaboration with arbuscular mycorrhizal fungi^16^.

Research on endophytic bacteria in cowpeas has revealed their potential to enhance plant growth and stress tolerance. For example, Omomowo and Babalola^17^ have identified and emphasized the diversity of endophytic strains carrying growth-promoting genes that improve seed germination and support plant growth under drought conditions. Another study demonstrated that *Bradyrhizobium elkanii* and the arbuscular mycorrhizal fungus *Rhizophagus irregularis* stimulated cowpea yield under drought conditions^18^. In addition, Muindi, et al.^19^ isolated and characterized rhizobial and non-rhizobial endophytic bacteria from cowpea, identifying certain strains that promoted cowpea growth.

Previous studies on endophytic bacteria have primarily focused on their role in mitigating drought impacts on cowpeas and promoting sustainable agricultural practices. However, research on the characterization and ecological dynamics of endophytic bacterial communities under drought conditions in cowpeas remains limited. Nonetheless, advances in next-generation sequencing (NGS) technologies have significantly enhanced our ability to understand endophytic microbiomes and the effects of drought on community structure^20^. Utilizing 16 S rRNA gene sequencing and amplicon sequence variant (ASV) analysis enables researchers to reveal the diversity, taxonomic profiles, and functional potential of endophytic bacterial communities associated with cowpeas under drought stress.

The purpose of this study was to comprehensively characterize the endophytic bacterial communities associated with cowpeas under optimal and drought-stressed conditions, and to provide a deeper understanding of the interactions between cowpeas and their endophytic bacteria. To achieve this purpose, a combination of culture-independent and culture-dependent methodologies was employed. The diversity and taxonomic composition of bacterial ASVs were analyzed, while endophytic bacteria with potential plant growth-promoting traits were isolated and characterized. The results showed that drought reduced microbial diversity and reshaped community composition, while several isolated endophytic bacteria displayed growth-promoting traits and increased wheat biomass under water stress. By integrating advanced molecular techniques with ecological insights, our work is thereby paving the way for the development of microbial inoculants tailored to enhance crop resilience in water-limited agroecosystems.

## Materials and methods

### Experimental design and plant growth conditions

The experiments aimed to investigate how drought influences the dynamics of endophytic bacterial communities associated with cowpea. Two treatment groups were established: a control group, in which cowpea plants were grown under optimal watering conditions, and a drought group, in which plants were subjected to drought stress by withholding water for 3 weeks. The study included four biological replicates per treatment group, with each replicate consisting of root samples collected from 4 cowpea plants. After the treatment period, the root tissues from each pot were pooled and considered as a biological replicate, and DNA was extracted for microbial analysis. High-throughput 16 S rRNA sequencing was performed on the DNA samples to identify bacterial communities. The cowpea growth conditions and drought treatments were conducted as previously described by Halo, et al. ^21^. Briefly, cowpea (*Vigna unguiculata* subsp. unguiculata) seeds were surface sterilized by immersing them in 70% ethanol for 3 min, followed by 40 s in a 10% bleach solution (6% sodium hypochlorite) with gentle agitation. They were then rinsed once with a 5% Tween-20 solution and twice with sterilized distilled water. Sterilized seeds were planted (four per pot) in eight 3-liter pots filled with approximately 2.8 L of soil collected from the Al Seeb area in Muscat, Oman (23.6472°N, 58.1456°E). To minimize contamination, pots and tools were washed with detergent, sterilized in 0.6% sodium hypochlorite, and rinsed with 75% ethanol followed by sterile water. The soil used in this study was previously analyzed^21^, where it was characterized as alkaline (pH 8.05) with low organic carbon (0.51% TOC), and containing 5.53% carbon, 0.66% hydrogen, 0.02% sulfur, and an electrical conductivity of 0.38 mS/cm. The pots were kept in a growth room at 24 °C with a 16-hour light/8-hour dark photoperiod, and all treatments received an equal amount of autoclaved destilled water to field capacity every 2–3 days for 6 weeks. Subsequently, the pots were divided into a control group (regular watering continued) and a drought group (no watering for 3 weeks). Nine weeks after planting, roots were harvested from four pots in each group, while soil water content and electrical conductivity (EC) were monitored using a 5TE sensor and ECH20 software (METER, Pullman, USA).

### DNA extraction

Root tissues from four plants per pot were pooled to form a biological replicate and surface-sterilized as previously described^22^. Initially, the attached soil of the roots was detached using sterilized forceps. The roots were then thoroughly washed with tap water to eliminate any remaining soil. Surface sterilization was performed by immersing the root tissues in 30% hydrogen peroxide for 3 min, followed by a 3-minute soak in 75% ethanol. The ethanol solution was discarded, and the tissues were then soaked in 0.6% sodium hypochlorite for 3 min. Following sterilization, the roots were washed once with sterile distilled water supplemented with 10% Tween-20, then three times with sterile distilled water. The surface-sterilized root tissues from each of the four biological replicates were gently dried with sterile tissue paper, and total DNA was then extracted from each replicate separately using the DNeasy Plant Mini Kit(Cat. No. 69104, Qiagen, Hilden, Germany) according to the manufacturer’s protocol. DNA quality and concentration were assessed using a Nanodrop 2000 spectrophotometer (Thermo Scientific, USA) and verified via 1% Tris-Acetate-EDTA (TAE) gel electrophoresis.

### 16 S rRNA gene sequencing metagenomic analysis

Eight sequencing libraries were prepared from DNA extracted from plant roots, each representing an independent biological replicate: four from control roots and four from drought-treated cowpea plants. Bacterial community analysis was performed via 16S rRNA amplicon sequencing targeting the V3–V4 region, using primers Bakt_341F (5’-CCTACGGGNGGCWGCAG-3’) and Bakt_805R (5’-GACTACHVGGGTATCTAATCC-3’). Sequencing was performed on an Illumina MiSeq platform using 300 bp paired-end reads across the eight libraries. Libraries were prepared using the Herculase II Fusion DNA Polymerase and Nextera XT Index V2 Kit following the Illumina 16 S metagenomic sequencing library preparation protocol (Part no. 15044223 Rev B). As a service company, sequencing was outsourced to Macrogen (Seoul, South Korea).

### Computational analysis of the sequenced DNA

Raw reads were processed, in which adapter and primer sequences were removed, and reads were trimmed to 250 bp (forward) and 200 bp (reverse) using Cutadapt (version 3.2). The DADA2 pipeline^23^ was used to generate Amplicon Sequence Variants (ASVs), in which reads were error-corrected, merged, and denoised. 16 S rRNA sequence length filtering was performed in R to retain biologically relevant reads, excluding organellar DNA. Reads with expected errors ≥ 2 were removed, and the remaining sequences were denoised using DADA2’s error model. After denoising, forward and reverse reads were joined based on their overlapping regions. Chimeric sequences were then identified and removed using the consensus-based removeBimeraDenovo function in DADA2. For taxonomic assignment, each ASV was matched to the most similar sequence in the NCBI_16S reference database using BLAST+ (v2.9.0) against a curated 16 S rRNA database (NCBI_16S_20231219), with thresholds of > 85% query coverage and > 85% identity. Subsequent processing and analysis of ASVs were carried out using QIIME (v1.9.0). Bacterial community alpha-diversity metrics, including the Shannon index, Gini-Simpson index, and phylogenetic diversity (PD) whole-tree, were calculated using QIIME (v1.9) (Caporaso et al., 2010) to assess species complexity within individual samples and across sample groups. The same software was used for microbial community comparison, in which sequencing depth was normalized by rarefying all samples to the read count of the lowest-depth sample. The resulting normalized ASV table was then used for downstream analyses. The Mafft (v7.475)^24^ and FastTreeMP (v2.1.10)^25^ were used to perform multiple alignments and construct phylogenetic trees of ASVs.

Beta diversity was assessed using principal coordinates analysis (PCoA) in PAST (version 3.0)^26^, and the Bray-Curtis metric. Differences in multivariate community composition among groups were tested using Permutational Multivariate Analysis of Variance (PERMANOVA)^27^, implemented within PAST. PERMANOVA evaluates whether group centroids differ significantly in multivariate space by partitioning the sums of squares of the distance matrix. The analysis was performed using 9,999 permutations, providing a robust estimate of significance. An F-statistic was computed and compared against the permuted distribution to obtain a *P*-value. Statistical significance was set at *P* < 0.05. Genetic relationships among samples were evaluated using the Unweighted Pair Group Method with Arithmetic Mean (UPGMA), a hierarchical clustering approach that uses average-linkage criteria on the distance matrix to visualize patterns of genetic relatedness^28^.

Differential abundance of each ASV between the two groups (control vs. drought treatment) was identified using an independent-samples t-test with statistical significance set at *P* < 0.05, and a phylogenetic tree was constructed in MEGA 11 using the Neighbor-Joining method, with the Kimura 2-parameter model, with 1,000 bootstrap replicates used to assess statistical support^29^.

### Isolation of endophytic plant growth-promoting bacteria (PGPB) and the ACC deaminase activity assay

Endophytic bacteria were isolated from drought-treated cowpea plants using standard culture methods, as previously described by Rashid, et al.^30^. Briefly, the root tissues were surface-sterilized using the protocol described earlier in this study. Then, ~ 5 g of the root tissues were homogenized in a sterile porcelain mortar with 10 mL of Ringer’s solution. The resulting extract was diluted tenfold with Ringer’s solution in sterile 15-ml Falcon tubes. Aliquots of 10–50 µL were spread onto King’s B agar, Luria Agar (LA), and Tryptic Soy Agar (TSA) plates. Plates were incubated at 32 °C and monitored daily for up to five days. Single colonies were re-streaked on the respective medium to ensure purity.

To isolate 1-aminocyclopropane-1-carboxylic acid (ACC) deaminase-producing bacteria, 1 mL of the plant root tissue Ringer’s extract was added to 50 mL of sterile *Pseudomonas* Agar F (PAF) medium in a 250-mL flask, and the flask was incubated at 30 °C in a shaking incubator (200 rpm). PAF medium facilitates the rapid isolation of ACC deaminase-producing PGPR by enriching beneficial bacteria and suppressing the growth of soil fungi^31^. The remaining protocol for bacterial selection and the ACC deaminase activity assay of the isolated PGPB was performed as described by Penrose and Glick^31^. Biochemical and genetic assays were performed on each isolate, and the functional characterization and identification of endophytic PGPB isolated from drought-treated plants were conducted using high-throughput protocols described by Yaish^32^.

### Ammonium production assay

The ammonium production capacity of the isolated PGPB was assayed using Nessler’s reagent as previously described^33^. PGPB isolates were cultured in 96-well plates containing TSB liquid medium until significant growth was observed (OD ≥ 1). Cultures were then transferred to 10-cm plates containing 1% proteose peptone medium and incubated at 30 °C with shaking at 210 rpm for 48–72 h. Nessler’s reagent (50 µL) was added to each well to detect ammonium production, with color changes recorded.

### Quantification of IAA and indole-like compounds

Indole-3-acetic acid (IAA) was quantified according to the protocol described by Gordon and Weber^34^. The measurements reflect approximate IAA levels, as other indole compounds may also be detected. PGPB strains were grown in deep 96-well plates containing tryptic soy broth (TSB) to mid-log phase (OD = 0.7). Cultures were then subcultured into Dworkin and Foster (DF) minimal medium^35^, supplemented with 500 µg/mL L-tryptophan, in deep 96-well plates with agitation at 210 rpm. DF minimal medium was used to minimize background interference and ensure IAA production is primarily induced by supplemented tryptophan^31^. A control culture in a deep 96-well plate was prepared without L-tryptophan. After 24 h of incubation at 30 °C with shaking, cells were centrifuged (4,000 rpm, 5 min, 4 °C). Supernatants (100 µL) of each sample were mixed with 100 µL of Salkowski’s reagent in a 96-well plate and incubated for 30 min in the dark, as previously described^36^. Absorbance was measured at 530 nm using a microplate reader spectrophotometer (Synergy MTX, multi-mode read, Biotek, Vermont, USA), and the relative color intensity for each strain was qualitatively evaluated using a “+” scoring system.

### Phosphorus, potassium, and zinc solubilization and siderophore production assays

Mineral solubilization assays were conducted on agar media containing insoluble salts, and the formation of a halo zone indicates microbial solubilization capacity. Therefore, Pikovskaya’s agar medium plates were supplemented with 0.15% Ca_3_(PO4)_2_ for the phosphorus solubilization assay. Bacterial strains were cultured overnight in LB medium, and 5-µL aliquots were spotted onto agar plates using the Pikovskaya protocol. Plates were incubated at 30 °C for 48 h, and halo zones indicating phosphorus solubilization were recorded. As previously described, Aleksandrow agar medium plates containing 0.2% mica as an insoluble potassium source were prepared for the potassium solubilization assay^37^. Bacterial strains grown in LB medium were spotted (5 µL) onto the agar plates. Plates were incubated at 30 °C for 48 h, and halo zones indicating potassium solubilization were recorded.

The zinc solubilization assay was performed according to Pikovskaya^38^ standard protocol. Pikovskaya’s agar medium plates supplemented with 0.15% ZnO were used to assess zinc solubilization. Bacterial strains were cultured overnight in a liquid LB medium and then spotted or streaked onto the agar medium. The plates were incubated at 30 °C for 48 h, and the halo zones were recorded. A chrome azurol S (CAS) agar assay was used to detect siderophore production, as previously described^39,40^. Bacterial strains were cultured overnight in LB medium, and single colonies were streaked onto CAS agar plates. Plates were incubated at 30 °C for 48 h, and siderophore production was indicated by yellow-orange zones surrounding the bacterial colonies. The relative halo diameter for each strain was qualitatively evaluated using a “+” scoring system.

## Screening for drought tolerance of the isolated strains

Screening bacterial tolerance to drought stress was conducted according to the procedure described by Niu, et al. ^41^. TSB medium was supplemented with 15% polyethylene glycol (PEG) 8,000 and inoculated with 1% exponentially grown bacterial cultures. The cultures were then incubated at 30 °C on a shaking incubator at 120 rpm. After 7 days, the growth rates of stressed and non-stressed cultures were evaluated by measuring the optical density at 600 nm using a spectrophotometer.

### Pot growth-promoting activity assay

The plant growth-promoting activity of the isolated bacterial strains was tested using a pot experiment as previously described, with minor modifications^42^. Briefly, selected isolated bacterial strains with high IAA production, ACC deaminase activity, and mineral solubilization capabilities were cultured in 50 mL TSB medium at 30 °C with agitation at 210 rpm for 24 h. Bacterial cells were harvested by centrifugation at 5,000 rpm for 3 min at 4 °C, washed with 0.85% NaCl, and then recentrifuged before discarding the supernatant. The cell pellet was resuspended in 0.85% NaCl to achieve an optical density at 600 nm (OD₆₀₀) of 0.25. Wheat (*Triticum aestivum*) seeds were surface-sterilized using ethanol and bleach solutions, as described above, and then coated with a bacterial suspension by soaking for 2 h at room temperature. Seeds in the no-bacteria treatment groups were soaked in a 0.85% NaCl solution to serve as a mock treatment. The experiment was conducted using a randomized design with three replicates per treatment. The soil was moistened with distilled water and autoclaved twice at 121 °C for 25 min before being used in this experiment, and all handling was carried out with sterile gloves and instruments. Seven wheat seeds were sown per treatment in sterile, moistened HAKO potting substrate (DE Lier, Netherlands) in 10 cm diameter sterile plastic pots. To minimize contamination, pots and tools were cleaned with detergent, surface-sterilized with 0.5% sodium hypochlorite, and then rinsed with 75% ethanol and sterile water. Bacterial inoculation was applied by direct deposition rather than spraying to reduce aerosol transfer. Irrigation was performed with autoclaved distilled water, and pots were spatially separated to limit cross-contamination.

The pots were divided into four groups: (1) bacteria-treated plants with regular irrigation, (2) bacteria-treated plants subjected to drought stress, (3) bacterial-untreated plants with regular irrigation, and (4) bacterial-untreated plants subjected to drought stress. Each seed in the bacterial-treatment pot received 1 mL of bacterial suspension (OD = 0.25), and each seed of the bacterial-untreated pot received 1 mL of 0.85% NaCl solution (mock solution), before all the seeds were covered with soil. Bacteria-treated plants were followed by a second bacterial treatment five weeks after planting. Plants were spaced to minimize cross-contamination, and both treatments were grown under the same clean conditions, ensuring equal exposure to any environmental contaminants. All wheat seedlings were irrigated uniformly across all treatments to provide consistent growth conditions. The plants were irrigated to field capacity with equal amounts of sterile water for 6 weeks to avoid both water stress and waterlogging. After this period, water was withheld from the drought-stress groups for approximately 2.5 weeks until most seedlings exhibited drought symptoms.

Meanwhile, the irrigated groups continued to receive water until they reached the desired growth stage. Consequently, the experiment lasted approximately 8.5 weeks. At the end of the experiment, seedlings were harvested, and biomass parameters, including plant height (shoot) and fresh and dry weights (shoot and root), were recorded and analyzed statistically as described below. Data were collected from the five most vigorous plants in each treatment. The dry weights of the plants were obtained by air-drying the seedlings in an oven at 65 °C for 48 h, as described by Halo et al. (2020). The dry and fresh weights were then measured using an electronic analytical scale (OHAUS, PR series, Nänikon, Switzerland). The relative water content of the soils in the pots at the end of the experiment was measured as described earlier in this report.

### Bacterial genome extraction, 16 S rRNA gene sequencing, and analysis

Each bacterial strain was grown in a 96-well deep culture plate containing 1 mL of 2x LB medium and incubated overnight at 30 °C with shaking at 210 rpm. Cells were harvested by centrifugation at 4,000 rpm for 15 min at 4 °C, washed twice with 1 mL of cold phosphate-buffered saline (PBS), and then resuspended in 1 mL of 1x PBS. Aliquots (100 µL) of the suspension were transferred to a 96-well PCR plate, lysed by freezing at ‒20 °C for 4 h, and heated to 96 °C for 15 min in a thermal cycler. Lysates were collected by centrifugation at 4,000 rpm for 20 min at 4 °C, and then 20 µl of the supernatant containing DNA was transferred to a new 96-well PCR plate.

DNA concentrations were quantified using a Nanodrop spectrophotometer (Thermo Scientific, USA). PCR amplification of the V1-V7 regions of the 16S rRNA gene was conducted using a 2x green master mix and primer pairs specific for bacterial identification, 27F 5’-AGAGTTTGATCCTGGCTCAG-3’ and 1492R 5’-GGTTACCTTGTTACGACTT-3’ ^43^. Amplified products were verified on 1% agarose gels, and PCR products were purified using a PCR purification kit (Thermo Scientific, USA). Finally, the PCR products were subjected to Sanger sequencing at Macrogen (South Korea). Sequences were analyzed to identify bacterial strains by performing a BLAST search against the National Center for Biotechnology Information (NCBI), USA, using the 16 S rRNA database, and the 16 S rRNA identity thresholds for the bacterial taxonomic units were assigned as previously described^44,45^. A phylogenetic tree was constructed from the 16 S rRNA gene sequences of the isolated strains using the method described previously in this report.

### Statistical analysis

Alpha-diversity metrics and differentially abundant ASVs between control and drought-treated cowpea plants were analyzed using independent-samples t-tests in SPSS v21.0 (IBM, 2012), with significance set at α = 0.05 (95% confidence interval). When Levene’s test indicated unequal variances, the “equal variances not assumed” results were applied, corresponding to Welch’s t-test with the Welch–Satterthwaite correction. Pairwise comparisons of wheat seedling biomass among treatments, following inoculation with cowpea-derived endophytic bacteria under control and drought conditions, were performed using one-way ANOVA followed by Fisher’s LSD, without additional multiple-comparison corrections.

## Results

### Deep sequencing of the 16 S rRNA gene and identification of ASVs

Sequencing of 16 S rRNA gene libraries from the roots of control and drought-treated cowpea plants generated a total of 733,880 reads across eight samples, with an average of 91,735 reads per sample. The sequencing underwent a series of filtration steps, including the exclusion of organellar sequences, to ensure high-quality data for analysis. These steps included adapter and primer trimming, length trimming, quality control, and ASV length filtration, ultimately refining the dataset to 544,431 reads, with an average of 68,054 reads per sample. The quality scores of the libraries (Q30/Q20 ratio) range from 0.89 to 0.91, which reflects high sequence quality. The sequencing of the four libraries for each treatment yielded an average of 72,958 ± SD = 1,735 and 63,149 ± SD = 7,750 filtered ASVs for the control (W) and drought-treated (D) plants, respectively. Statistical analysis revealed no significant difference in total ASVs between the two groups (*P* > 0.05).

The processed data revealed 276 amplicon sequence variants (ASVs), including 47 unclassified variants (Table S1). The ASV lengths ranged from 382 to 428 bp, with an average length of ~ 414 bp. Taxonomic annotation classified the ASVs into eight phyla (Actinomycetota, Bacillota, Bacteroidota, Cyanobacteriota, Myxococcota, Planctomycetota, Pseudomonadota, and Verrucomicrobiota), 17 classes, 45 orders, 65 families, 102 genera, and 154 species, representing 336,462 abundance classified and 106,994 unclassified ASVs. Among the eight libraries, the phylum Cyanobacteriota was the most abundant, with Cyanophyceae identified as the dominant class. The order Nodosilineales showed the highest prevalence, and the family Nodosilineaceae was the most prominent. At the genus level, *Marileptolyngbya* was the most abundant, while the cyanobacteria species *Marileptolyngbya sina*, followed by the nitrogen-fixing bacterium Ensifer aridi, exhibited the highest representation.

### Drought decreased species richness and evenness within endophytic bacterial communities

**Fig. 1:**
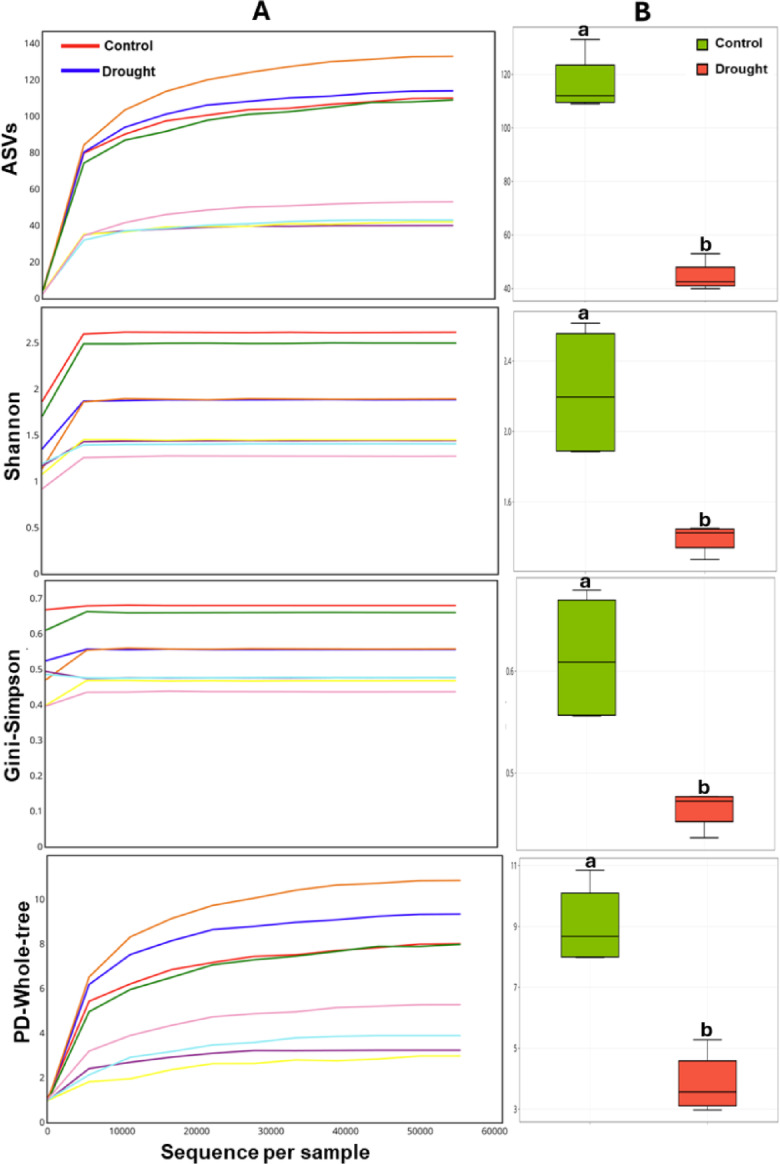
Alpha diversity of endophytic rhizobacteria associated with cowpea roots under control and drought conditions. (**A**) Rarefaction curves showing normalized sequencing depth and ASV richness in bacterial communities across treatments assessed using multiple alpha diversity indices. (**B**) Box plots display alpha-diversity metrics for the two treatments, with statistical differences assessed using independent-samples t-tests (*P* ≤ 0.05; α = 0.05, 95% confidence interval).

Alpha diversity analysis was performed to evaluate structural variations within microbial communities under control and drought treatments, employing rarefaction-based methods and overall alpha diversity indices. Based on the number of sequences from both groups, the rarefaction curves provided insights into microbial diversity within cowpea endophytic bacterial communities, enabling fair comparisons across samples with varying sequencing depths and a balanced assessment of microbial richness and evenness under different conditions. The comparative rarefaction-alpha diversity analysis of all measured indices, including ASVs, Shannon, Gini-Simpson, and PD-Whole-tree index, revealed that drought-treated samples exhibited significantly lower diversity (*P* ≤ 0.05) (Fig. 1A). Specifically, the ASV and Shannon indices showed a significant (*P* ≤ 0.05) decrease in microbial diversity and evenness in the drought group compared with the controls, reflecting lower richness of unique taxa and a less balanced microbial community. Phylogenetic diversity (PD-Whole Tree) analysis further confirmed reduced phylogenetic diversity under drought, suggesting a narrower range of evolutionarily distinct taxa. Interestingly, the Gini-Simpson index indicated lower species dominance in both the drought and control samples, suggesting lower abundance distributions of the most common species.

The overall alpha-diversity analysis revealed a significant (*P* ≤ 0.05) reduction in endophytic microbial community diversity under drought conditions (Fig. 1B). This decline was consistently observed across multiple diversity metrics, including ASV, Shannon, Gini-Simpson, and PD-whole tree indices.

### Drought reduced diversity among endophytic bacterial communities

Beta diversity analysis was performed to assess how drought influences overall community composition and microbial assemblage patterns and to evaluate replicate consistency using PCoA based on species abundance data from the four replicates in each group.

**Fig. 2:**
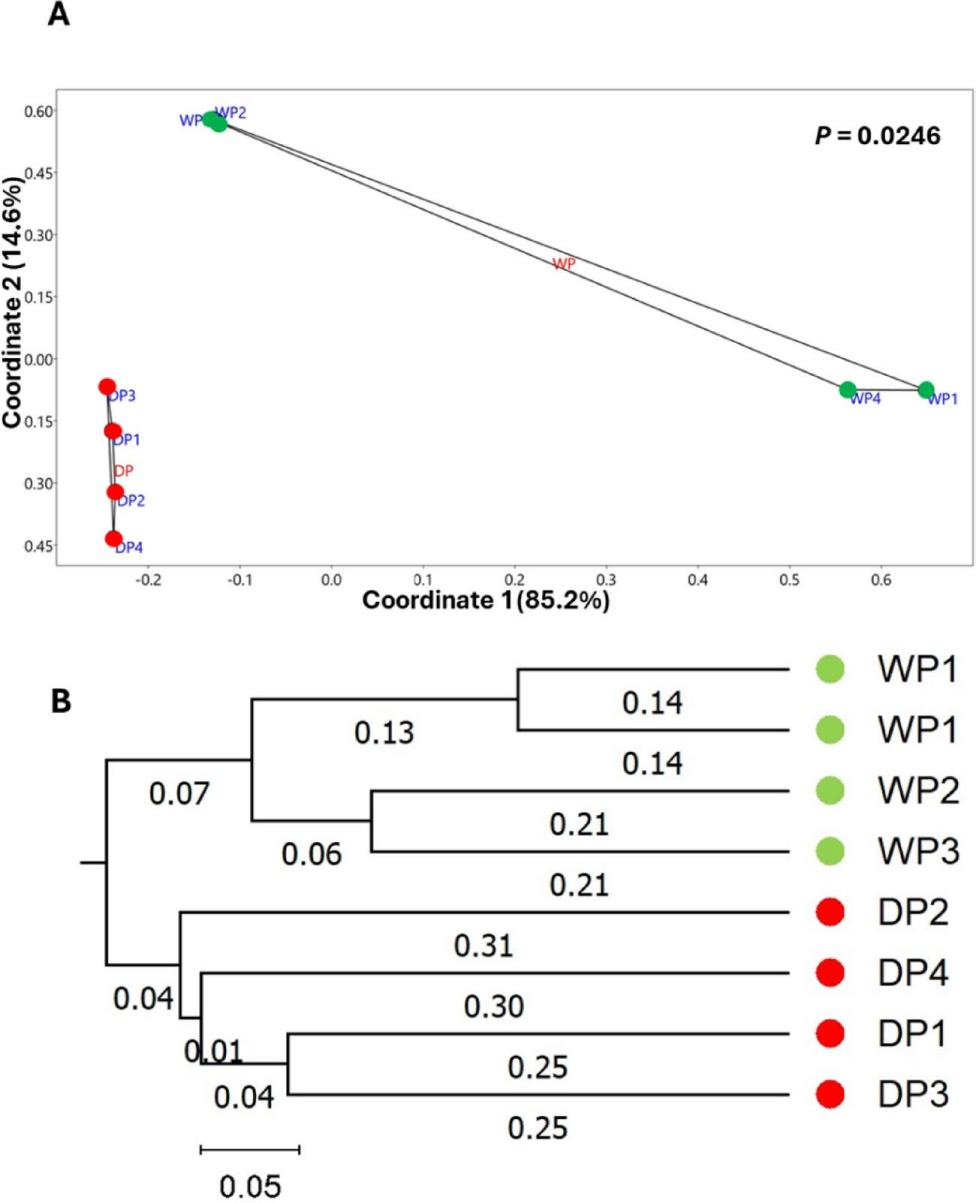
Beta diversity analysis of bacterial species from eight independent endophytic rhizobacterial communities associated with cowpea plants grown under control (WP1–WP4) and drought (DP1–DP4) conditions, based on species abundance. (**A**) The principal coordinates analysis (PCoA) explains 85.2% and 14.6% of the total variation in the first and second coordinates, respectively. The PERMANOVA *P*-value on the PCoA plot indicates a significant separation between the control and the treated groups. (**B**) The UPGMA phylogenetic tree, generated using the Unweighted UniFrac distance metric, illustrates the genetic relationships among samples. WP samples and DP samples form two distinct clusters. Numbers on the branches indicate the corresponding distance values, and the scale bar represents the genetic distance used to construct the tree.

Using the Bray–Curtis distance matrix, the PCoA explained about 99.8% of the total variation in the samples and showed that most of the variation in community composition was captured by the first two axes, with PCoA1 explaining 85.2% and PCoA2 explaining 14.6%. The endophytic bacterial communities from drought-treated plants formed a tightly clustered group, indicating a high degree of similarity among replicates. In contrast, the bacterial communities from control plants exhibited higher dispersion, indicating greater variability in community composition. To test whether the observed clustering reflected fundamental group differences, a PERMANOVA (9999 permutations) was performed, yielding an F value of 5.845 and a *P*-value of 0.0246. These results indicate statistically significant differences among the drought-treated and control groups, confirming that the multivariate patterns observed in the PCoA are not due to random variation and that drought drives shifts in endophytic bacterial composition (Fig. 2A).

To confirm this result, a dendrogram generated using the UPGMA clustering method shows a clear phylogenetic separation between the control (WP) and drought (DP) groups, with each forming a distinct, cohesive cluster. The branch lengths separating the two clusters were notably longer than those within clusters, indicating greater genetic divergence between groups than among samples within the same group (Fig. 2B). These clustering patterns align with the PERMANOVA results, further supporting significant group-level differentiation. These results highlight the considerable impact of drought on microbial community composition, with drought-treated samples showing more homogeneous biodiversity.

### Drought reshapes cowpea endophytic bacterial diversity

Drought significantly alters the composition of endophytic bacterial communities in cowpea plants, leading to changes in taxonomic diversity and species abundance. Statistical and taxonomic analyses highlighted key microbial groups that respond to drought, providing insights into their potential roles in adaptation to water stress (Tables S1 and S2).

**Fig. 3:**
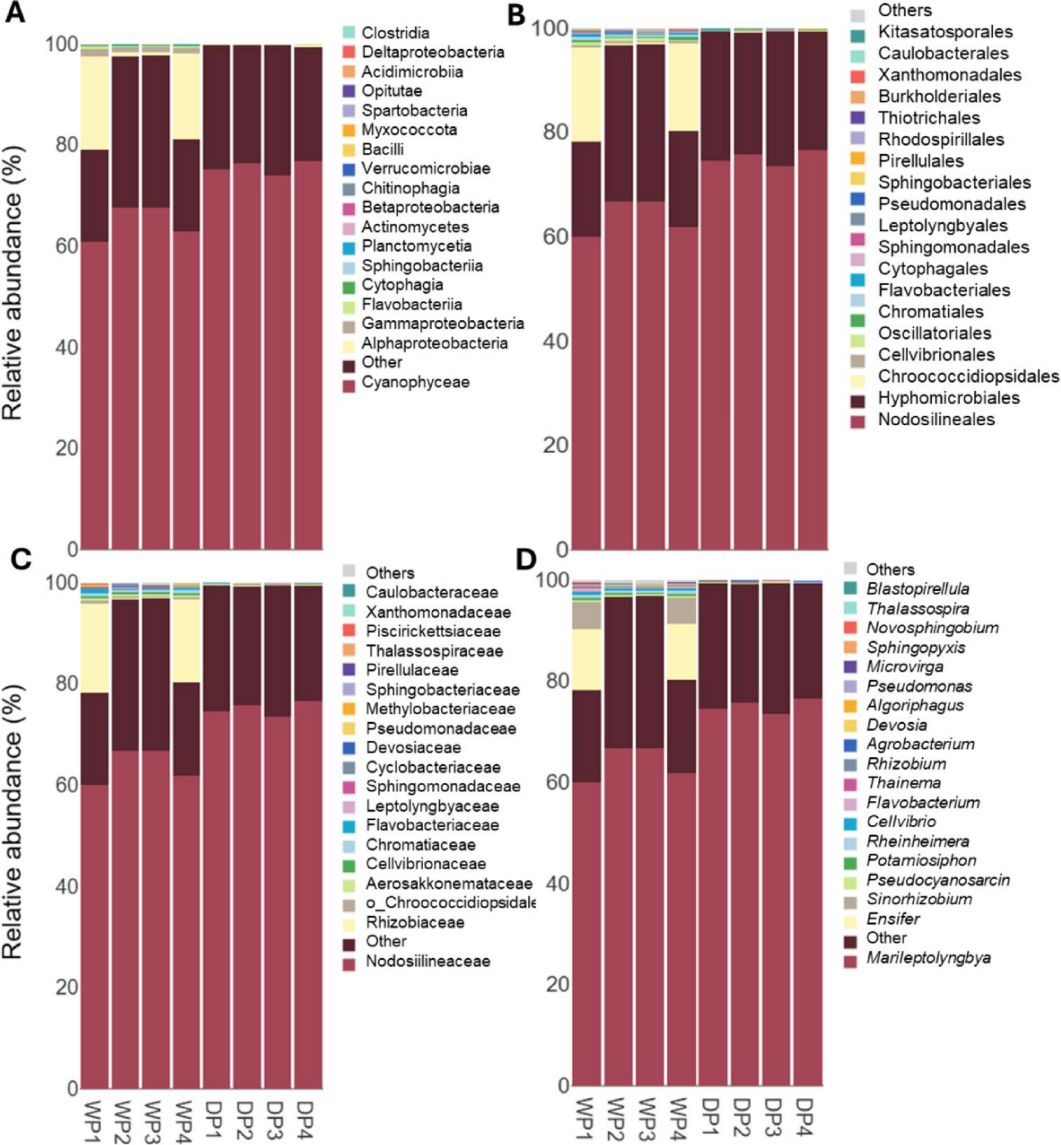
The relative taxonomic abundance of the top 20 taxa derived from amplicon sequence variants (ASVs) at the class (**A**), order (**B**), family (**C**), and genus (**D**) levels within the endophytic microbial communities identified in cowpea plants grown under control (WP1–WP4) and drought (DP1–DP4) conditions, where WP1–WP4 and DP1–DP4 represent four biological replicates per treatment.

Taxonomic annotation of the ASVs and relative abundance revealed significant (*P* ≤ 0.05) changes in bacterial community composition under drought conditions. Cyanobacteriota was the only phylum to show significantly higher abundances (*P* ≤ 0.05) in the endophytic bacterial communities of cowpea plants grown under drought conditions compared to controls (Table S2). Among the bacterial classes, Gammaproteobacteria, Cytophagia, Cyanophyceae, Actinomycetes, and Planctomycetia were differentially abundant, with Cyanophyceae being the only class significantly (*P* ≤ 0.05) enriched under drought conditions (Fig. 3A). The analysis also revealed that 13 orders and 12 families were differentially accumulated at a significant level (*P* ≤ 0.05) in response to drought conditions in cowpea plants, with Nodosilineales at the order level and Nodosilineaceae at the family level exhibiting significantly higher abundance under the drought treatment (Fig. 3B and C). Seventeen bacterial genera were significantly (*P* ≤ 0.05) accumulated in response to drought, including Marileptolyngbya, which was higher in cowpeas exposed to drought (Fig. 3D).

The taxonomic and abundance analysis of 154 identified endophytic bacterial species revealed that 19 bacterial ASVs showed significant (*P* ≤ 0.05) differences in abundance within the cowpea endophytic community under drought conditions (Table S2). Only one bacterial species, *Marileptolyngbya sina*, a cyanobacterium, was significantly enriched under drought conditions. However, *Potamosiphon australiensis*, *Pseudocyanosarcina phycocyania*, and *Thainema salinarum*, also classified as cyanobacterium endophytic ASVs, accumulated to a substantially greater extent under control conditions in cowpea roots (Table 1).

**Table 1:** Differentially accumulated endophytic bacteria in cowpea under control and drought conditions. Endophytic bacterial taxa exhibiting significant differences in abundance were identified based on average abundance across four ASV libraries in plants grown under control (WP) and drought (DP) conditions (values are presented as mean ± SD). Differences between groups were assessed using independent-samples t-tests, with a significance level set at α = 0.05 (95% confidence interval).

ASV	Mean ± SD (WP)	Mean ± SD (DP)	*P*-Value
*Rhizobium helanshanense*	44.75 ± 4.99	3.25 ± 3.95	0.0000
*Cellvibrio diazotrophicus*	253.25 ± 40.8	13.50 ± 10.91	0.0000
*Echinicola sediminis*	15.75 ± 3.3	0.00 ± 0	0.0001
*Novosphingobium arabidopsis*	29.75 ± 5.12	1.50 ± 3	0.0001
*Methylophaga pinxianii*	17.00 ± 2.94	0.00 ± 0	0.0014
*Rheinheimera pleomorphica*	276.75 ± 62.44	4.75 ± 6.18	0.0030
*Marileptolyngbya sina*	35370.25 ± 1927.44	41641.50 ± 748.77	0.0041
*Potamosiphon australiensis*	206.25 ± 25.99	78.25 ± 48.16	0.0067
*Blastopirellula marina*	23.25 ± 7.5	1.00 ± 2	0.0073
*Fulvivirga kasyanovii*	4.75 ± 2.5	0.00 ± 0	0.0090
*Sphingopyxis solisilvae*	23.00 ± 8	0.00 ± 8	0.0104
*Thainema salinarum*	65.00 ± 3.37	28.75 ± 19.59	0.0107
*Devosia salina*	17.25 ± 6.55	0.00 ± 0	0.0133
*Brevundimonas canariensis*	15.00 ± 9.93	0.00 ± 0	0.0234
*Pseudocyanosarcina phycocyania*	227.00 ± 28.37	137.50 ± 56.01	0.0291
*Rhizobium rosettiformans*	17.00 ± 4.69	5.50 ± 6.81	0.0319
*Algoriphagus aestuariicola*	37.00 ± 19.58	1.75 ± 3.5	0.0346
*Flavipsychrobacter stenotrophus*	8.50 ± 4.65	0.00 ± 0	0.0354
*Rickettsiella massiliensis*	2.00 ± 1.63	0.00 ± 0	0.0498

**Fig. 4:**
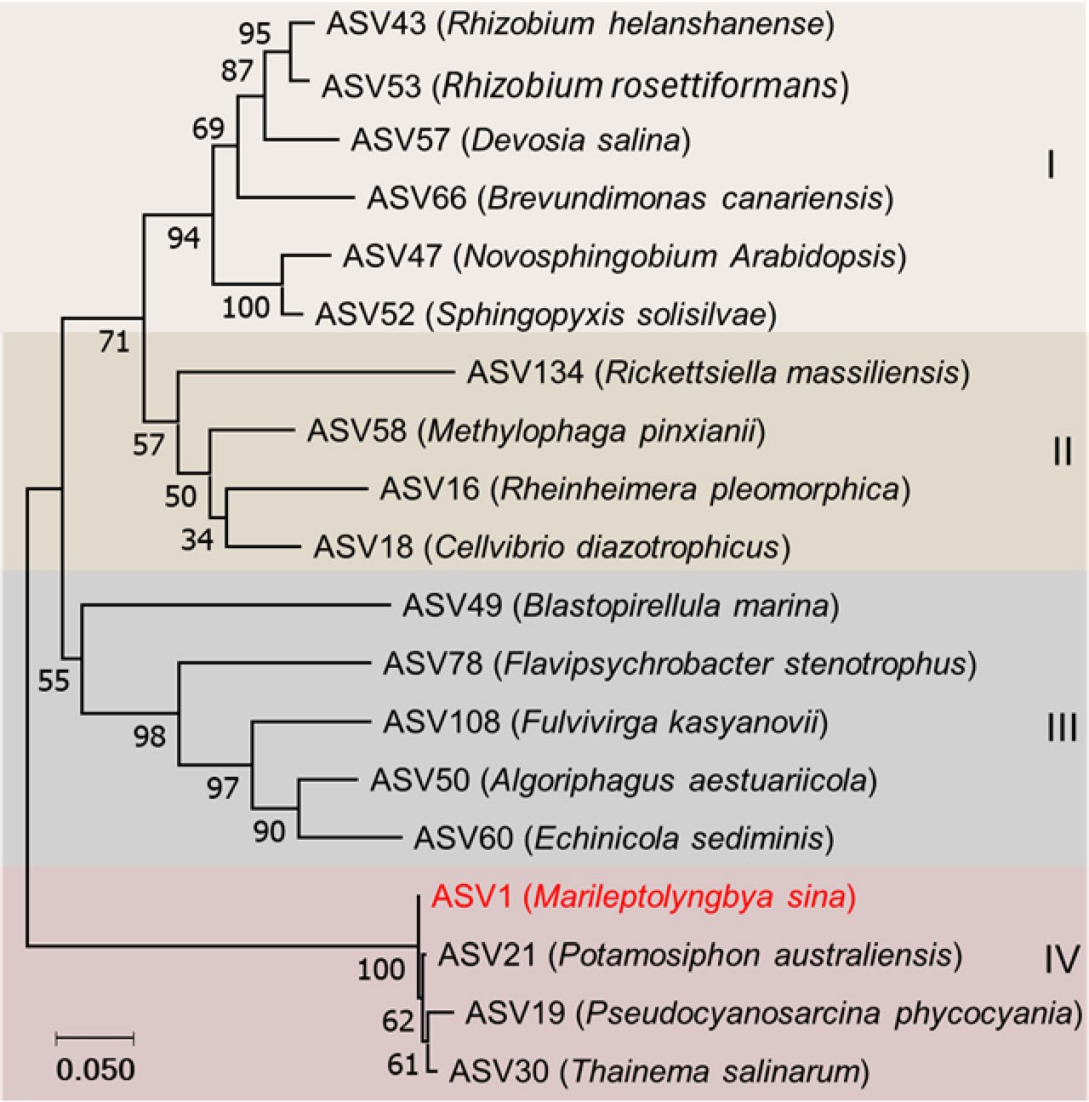
Phylogenetic relationships among differentially accumulated amplicon sequence variants (ASVs) in response to drought (significance set *P* ≤ 0.05) from cowpea roots. The tree was constructed using the Neighbor-Joining method, and bootstrap values represent percentage support from 1000 bootstrap replicates. The enriched strain in response to drought is highlighted in red. The analysis revealed four distinct cluster groups (I–IV). The scale bar represents genetic distance among the ASV sequences.

Phylogenetic analysis of the 16 S rRNA genes from differentially abundant ASVs revealed four distinct clades (I, II, III, IV), each representing distinct bacterial lineages with potential ecological roles (Fig. 4). Clade I, which includes *Rhizobium*, *Devosia*, *Brevundimonas*, *Novosphingobium*, and *Sphingopyxis* genera, is associated with nitrogen fixation. Clade II, comprising the genera *Rickettsiella*, *Methylophaga*, *Rheinheimera*, and *Cellvibrio*, is characterized by diverse metabolic capabilities, including methylotrophy and nitrogen cycling. In contrast, clade III, comprising *Blastopirellula*,* Flavipsychrobacter*,* Fulvivirga*,* Algoriphagus*, and *Echinicola* genera, is dominated by marine bacteria that typically degrade organic matter. Finally, clade IV, encompassing *Potamosiphon*, *Pseudocyanosarcina*, and *Thainema*, included cyanobacteria species that usually play a crucial role in photosynthesis.

### Isolation and phylogenetic analysis of the endophytic bacteria

The culture-based method enabled the isolation of viable endophytic bacteria for functional characterization and potential agricultural applications. Therefore, endophytic strains were isolated and characterized from cowpea roots using two strategies. The first strategy employed selective media for ACC deaminase-producing bacterial strains. This method yielded 17 strains, categorized into the first group, comprising VU-E1 to VU-E13, VU-E37, VU-E43, VU-E44, and VU-E49 strains. The second strategy involved extracting bacterial strains from root tissues and then directly streaking them onto rich agar media with varied components without considering their ability to produce ACC deaminase. This approach yielded 30 strains, designated the second group, comprising the remaining strains.

Identification of bacterial strains by 16 S rRNA gene sequence analysis revealed that the two methods yielded distinct bacterial community compositions. The first group was predominantly comprised of strains classified within Enterobacterales, with the majority belonging to the Enterobacteriaceae family, representing their lowest original shared taxonomic unit. In contrast, the second group isolates exhibited a broader taxonomic distribution, with their lowest standard taxonomic unit at the phylum level (Pseudomonadota/Proteobacteria), while the class Gammaproteobacteria was the most prevalent. Notably, cyanobacteria were not detected under the culture conditions used in this study (Table S3).

**Fig. 5:**
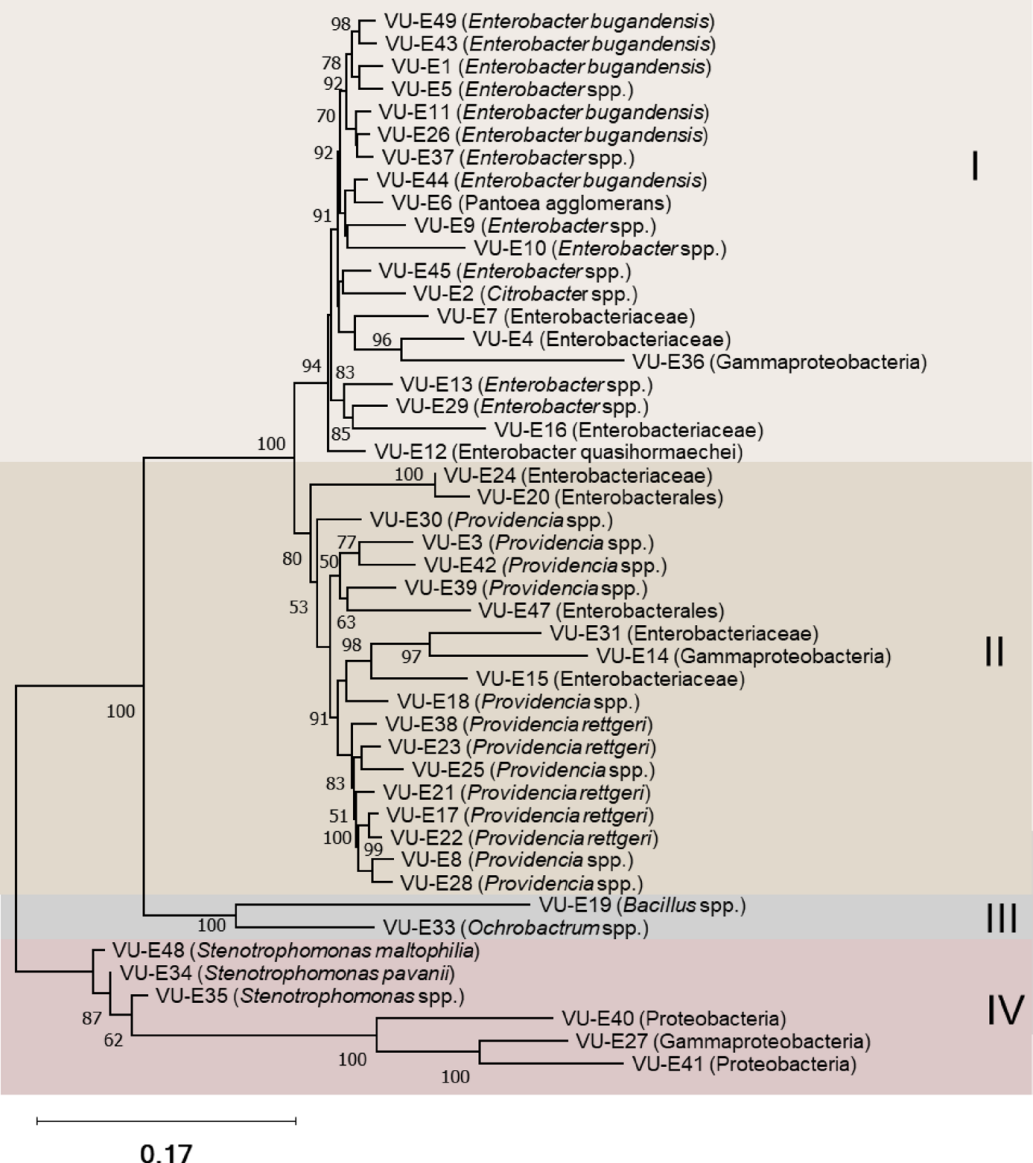
Phylogenetic relationships among the isolated endophytic bacteria from cowpea roots based on 16 S rRNA gene sequence analysis. The bootstrap values (≥ 50%) represent the percentage support from 1000 replicates constructed using the Neighbor-Joining method. The phylogenetic analysis identified four distinct cluster groups (I–IV). The scale bar represents genetic distance among the 16 S rRNA gene sequences.

Phylogenetic analysis of 16 S rRNA gene sequences revealed four distinct clades (I–IV). Clade I predominantly comprised strains belonging to *Enterobacter*, while clade II consisted of species from *Providencia*. Clade III encompassed genera *Bacillus* and *Ochrobactrum* members, whereas Clade IV included strains of *Stenotrophomonas* and representatives of the class Gammaproteobacteria (Fig. 5). Interestingly, most bacterial strains (15 out of 17) isolated using ACC deaminase-selective media clustered within clade I of the phylogenetic tree, alongside other strains isolated by different methods.

### Biochemical characterization and osmotolerance of endophytic strains

Endophytic bacteria were characterized for key biochemical traits related to plant growth promotion, including stress tolerance, nutrient mobilization, and hormone regulation (IAA synthesis and ammonium (NH₄⁺) production), ACC deaminase activity, and tolerance to 15% PEG, indicating drought resistance. Strains were also tested for their ability to produce siderophores for Fe³⁺ acquisition and to enhance the solubilization of potassium, phosphate, and zinc from mineral-bound forms, thereby increasing their bioavailability to plants (Table 2). The results showed that strains VU-E3, VU-E9, VU-E26, and VU-E47 exhibited the highest biological activities, as measured by the number of positive traits, making them strong candidates for enhancing plant growth.

PEG (15%) tolerance, which indicates resilience to osmotic stress and drought conditions, was highest in strains VU-E33, VU-E34, VU-E35, VU-E41, and VU-E44. VU-E25, VU-E26, VU-E28, VU-E35, VU-E36, and VU-E47 exhibited the highest IAA and other indole-like compounds levels. At the same time, strains VU-E24, VU-E26, VU-E39, and VU-E45 exhibited the highest ACC deaminase activity, indicating their effectiveness in mitigating plant stress by reducing ethylene levels. Additionally, siderophore production (Fe-Sid) was most pronounced in strains VU-E2, VU-E6, VU-E8, VU-E9, VU-E12, VU-E13, VU-E22, VU-E27, and VU-E47, making these strains particularly effective at iron acquisition, an essential factor for chlorophyll synthesis and plant health. For nutrient solubilization, the highest level of phosphate (P) solubilization was observed in strains VU-E3, VU-E21, VU-E29, and VU-E47, while zinc (Zn) solubilization was highest in strains VU-E18 and VU-E27. Potassium (K) solubilization, which aids enzyme activation and water balance, was most significant in strains VU-E1, VU-E3-8, VU-E18, VU-E27, and VU-E39. Furthermore, ammonium (NH₄⁺) production, which contributes to nitrogen availability for plant growth, was recorded in all strains except strain VU-E48.

**Table 2:** Tolerance of endophytic bacterial strains isolated from cowpea roots to 15% polyethylene glycol (PEG) and key plant growth-promoting traits, including indole-3-acetic acid (IAA) production, 1-aminocyclopropane-1-carboxylate (ACC) deaminase activity, siderophore production (Fe-Sid), ammonium (NH₄⁺) production, and the solubilization of phosphate (P), zinc (Zn), and potassium (K). Levels of each activity are indicated as: “+” (low), “++” (moderate), “+++” (high), and “++++” (extremely high), while “-” denotes absence of the trait.

Endophytic Strain	PEG (15%)	IAA	ACC deaminase	Fe-Sid	*P*	Zn	K	NH₄⁺
VU-E1	++	+	-	-	-	+	+	++
VU-E2	++	+	++	+	-	+	-	+
VU-E3	++	+	-	-	+++	++	+	++
VU-E4	++	+	++	-	-	+	+	++
VU-E5	++	+	-	-	+	+	+	+
VU-E6	++	+	-	+	++	+	+	++
VU-E7	+++	-	++	-	-	++	+	+
VU-E8	+	+	-	+	-	+	+	+
VU-E9	+++	+	++	+	+	+	-	++
VU-E10	+	+	-	-	+	+	-	++
VU-E11	+	+	+	-	-	+	-	+
VU-E12	++	-	++	+	++	+	-	++
VU-E13	++	++	+	+	-	+	-	+
VU-E14	++	-	++	-	-	+	-	+
VU-E15	++	-	++	-	+	++	-	++
VU-E16	++	+	++	-	-	-	-	+
VU-E17	++	+	++	-	-	-	-	+
VU-E18	+	-	++	-	-	+++	+	++
VU-E19	++	+	+	-	+	-	-	++
VU-E20	++	++	-	-	-	-	-	+
VU-E21	++	-	+	-	+++	-	-	+
VU-E22	++	-	+	+	-	-	-	+
VU-E23	++	-	+	-	-	+	-	++
VU-E24	++	++	+++	-	+	-	-	+
VU-E25	++	+++	-	-	-	-	-	+
VU-E26	++	+++	+++	-	+	-	-	++
VU-E27	++	+	-	+	+	+++	+	+
VU-E28	++	+++	-	-	+	-	-	++
VU-E29	++	-	-	-	+++	-	-	+
VU-E30	+++	++	++	-	-	-	-	+
VU-E31	++	+	++	-	-	-	-	+
VU-E33	++++	-	+	-	-	+	-	+
VU-E34	++++	++	-	-	-	-	-	++
VU-E35	++++	+++	-	-	-	-	-	++
VU-E36	++	+++	+	-	+	-	-	-
VU-E37	+++	+	-	-	+	+	-	+
VU-E38	+	+	+	-	-	+	-	+
VU-E39	++	-	+++	-	-	++	+	+
VU-E40	++	+	-	-	++	-	-	++
VU-E41	++++	+	-	-	-	+	-	+
VU-E42	+++	++	-	-	-	-	-	+
VU-E43	++	+	-	-	-	+	-	++
VU-E44	++++	+	++	-	-	++	-	+
VU-E45	++	-	+++	-	-	-	-	+
VU-E46	++	+	-	-	++	-	-	++
VU-E47	+++	+++	-	+	+++	-	-	+
VU-E48	+++	++	-	-	-	-	-	-
VU-E49	++	+	-	-	+	+	-	++

### Endophytic bacteria from cowpeas enhance the biomass of wheat seedlings

**Fig. 6:**
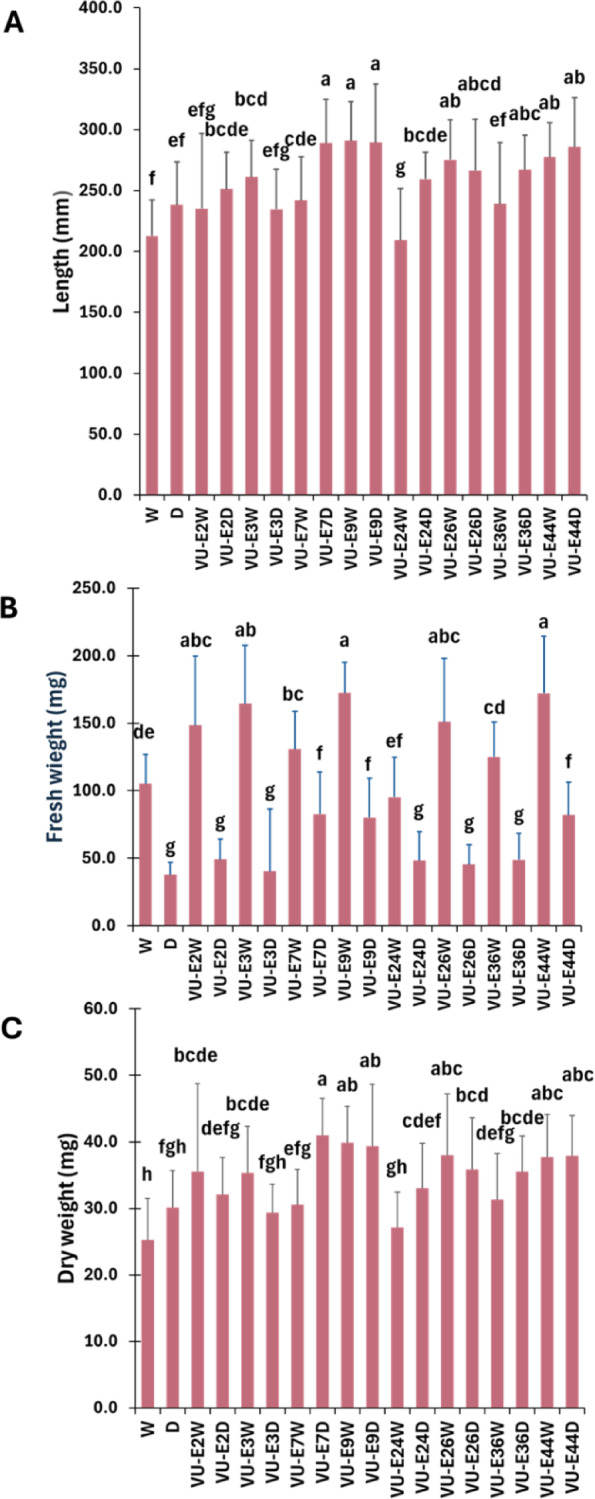
Effects of inoculation with endophytic bacteria isolated from cowpea on wheat seedling biomass under control and drought conditions. Seedling length (**A**), fresh weight (**B**), and dry weight (**C**). Bars represent mean ± SD. W and D represent uninoculated control plants grown under well-watered and drought conditions, respectively. Strain identifiers in the graphs ending in “W” correspond to control (well-watered) conditions, whereas those ending in “D” correspond to drought conditions. Different letters indicate significant differences among treatments (one-way ANOVA followed by Fisher’s LSD test, α = 0.05).

To assess the growth-promoting potential of the isolated strains, wheat seeds were inoculated individually with eight selected bacterial strains (VU-E2, VU-E3, VU-E7, VU-E9, VU-E24, VU-E26, VU-E36, and VU-E44) in pot assays. The biomass parameters, including shoot length and the fresh and dry weights of shoot and root tissues, were measured under both control and drought conditions. The results indicated that, under control conditions, all tested bacterial strains, except VU-E2 and VU-E36, significantly (*P* ≤ 0.05) increased seedling length compared to non-inoculated plants; however, under drought conditions, inoculation with strains VU-E7, VU-E9, VU-E26, VU-E36, and VU-E44 significantly enhanced seedling length compared to non-inoculated stressed plants (Fig. 6A). The results also demonstrated that, under control conditions, all tested bacterial strains, except VU-E24 and VU-E36, significantly (*P* ≤ 0.05) increased seedling fresh weight compared to non-inoculated plants. However, under drought conditions, only inoculation with strains VU-E7, VU-E9, and VU-E44 significantly enhanced seedling fresh weight compared to the non-inoculated stressed treatment (Fig. 6B). The results further demonstrated that, under control conditions, all tested bacterial strains, except VU-E24, significantly (*P* ≤ 0.05) increased seedling dry weight compared to non-inoculated plants. However, under drought conditions, inoculation with strains VU-E7, VU-E9, VU-E26, VU-E36, and VU-E44 significantly enhanced seedling dry weight compared to the non-inoculated control treatment (Fig. 6C). Therefore, strains VU-E7, VU-E9, and VU-E44 consistently enhanced wheat biomass under both control and drought conditions, showing improvements in seedling length, fresh weight, and dry weight. Based on the above results, these strains show a potential to promote wheat growth and stress resilience under field conditions.

## Discussion

Despite their inherent tolerance to environmental stress, cowpea plants exhibit significant reductions in multiple growth parameters under drought conditions^5,21^. This finding is consistent with the limited capacity of cowpea, like many other plant species, to tolerate drought stress^46^. Nevertheless, rhizospheric bacterial communities play a crucial role in enhancing plant drought resilience by improving soil properties and supporting plant viability^47^. However, their biodiversity and ecological functions remain insufficiently characterized^48^. Because cowpea cultivars exhibit only modest inherent drought tolerance, particularly under severe water deficits, identifying root endophytic bacterial communities and assessing their role in drought mitigation remains an important priority. Addressing this priority requires qualitative and quantitative analyses using high-throughput next-generation sequencing (NGS) technologies, combined with the isolation and characterization of endophytic plant growth-promoting strains from stressed plant roots. Insights gained from this research may help elucidate the key bacterial contributors to drought tolerance mechanisms in cowpea, with potential applications in the cultivation and commercialization of microbial inoculants to enhance drought resistance in various crops^49^.

In this project, the endophytic bacterial communities of cowpea roots grown under control and drought conditions were characterized. The results indicated that, unlike epiphytic rhizospheric bacteria, which showed increased diversity under drought conditions in cowpea plants^21^, the drought stress reduced the diversity of endophytic bacterial communities within the plant and across microbial populations. Diversity within each endophytic bacterial community decreased due to drought, as indicated by consistent declines across multiple diversity metrics, including ASV richness, Shannon, Gini-Simpson, and PD-whole-tree indices. The decrease in ASV richness reflected reduced species variability, while the Shannon and Gini-Simpson indices indicated lower species richness and evenness, suggesting that fewer drought-tolerant taxa became dominant. The reduction in PD-whole-tree values indicated a contraction in phylogenetic diversity within the endophytic bacterial community, reflecting decreased evolutionary distinctiveness among taxa under drought conditions. These findings suggest that drought exerts a selective effect on community composition, favoring specific microbial lineages and simplifying community structure^50^.

Contrary to our findings, in rice, drought stress increased the Shannon diversity index of endophytic bacterial communities, indicating a higher level of bacterial diversity in the endosphere under drought conditions, where the bacterial community structure shifted and specific taxa, such as Actinobacteriota and Gemmatimonadetes, were enriched^51^. These findings suggest that drought stress can lead to the selection of specific bacterial taxa better adapted to drought conditions. Soil moisture is a key driver of microbial diversity, and drought conditions can favor the proliferation of stress-tolerant bacterial species while suppressing those sensitive to water scarcity^52^.

Drought stress significantly reduces microbial diversity within plant-associated bacterial communities by altering root exudation patterns, reducing microbial colonization, and shifting taxonomic dominance^53^. For example, in soybean plants, significant differences in endophytic microbial community structure were observed between surviving and non-surviving plants under drought stress. Proteobacteria, *Pseudomonas*, and *Pantoea* dominated the surviving roots, while Streptomyces was predominant in the non-surviving roots, indicating drought-driven shifts in microbial community composition^54^. Changes in microbial community structures in sorghum have demonstrated that drought conditions can hinder early root microbiome development while promoting the abundance and activity of monoderm bacteria^55^.

Comparison of the differentially accumulated endophytic communities identified in this study with previously characterized epiphytic communities from the same cowpea plants^21^, revealed a limited number of shared ASVs during drought. *Rhizobium helanshanense* was the only species common to both communities, and its abundance declined markedly under drought. At the genus level, *Rhizobium*, *Methylophaga* spp., and *Devosia* spp. were shared across endophytic and epiphytic communities. *Rhizobium* spp. showed a consistent drought-induced reduction in both communities. *Methylophaga* spp. decreased within the endophytic compartment but increased in the epiphytic community under drought. In contrast, *Devosia* spp. accumulated more abundantly under control conditions in both compartments, suggesting a potential drought-responsive or drought-tolerant role. The shared microbial taxa that exhibited parallel shifts in both endophytic and epiphytic compartments under drought conditions indicate conserved microbial responses within cowpea, suggesting that these microorganisms may play essential roles in drought adaptation^53^.

The nitrogen-fixing bacterium *Ensifer aridus* was among the cowpea plants’ most abundant endophytic bacterial species. This bacterium enhances legume growth and soil fertility in saline and semiarid regions^56^, as it is adapted to harsh, arid environments, utilizing specialized metabolic pathways for stress resilience and effective plant symbiosis^57,58^. The abundance of the Cyanobacteriota phylum was significantly higher in cowpea endophytic bacteria under drought conditions compared to control conditions. Notably, the cyanobacteria species *Marileptolyngbya sina* exhibited a significant increase under drought stress. In contrast, the other phylogenetically related cyanobacteria species identified in this study, *Potamosiphon australiensis*, *Pseudocyanosarcina phycocyania*, and *Thainema salinarum*, did not show a similar accumulation. This observation may suggest functional divergence within the Cyanobacteriota phylum, likely driven by species-specific genetic adaptations that confer greater drought resilience in stressed environments^59^. *Marileptolyngbya sina*, which was isolated from Xincun Bay and the northern portion of the South China Sea, is a filamentous cyanobacterium involved in nitrogen fixation, contributing to marine nutrient cycling and primary production; however, its role in cowpea plants’ drought tolerance has not yet been explored^60^.

Functional prediction suggests that cyanobacteria from the *Leptolyngbyaceae* family, including *Marileptolyngbya sp.*, may enhance plant growth and soil resilience under drought through multiple genomic and metabolic mechanisms, according to the available literature. Genomic studies of related species such as *Leptolyngbya sp. NIES-2104* reveals genes associated with trehalose and mycosporine-like amino acid (MAA) synthesis, which protect against UV and desiccation stress^61^. These cyanobacteria also produce carotenoids and antioxidants that mitigate oxidative damage^62^, and secrete exopolysaccharides (EPS) that enhance soil aggregation and water retention^63^. Metabolomic analyses show that related cyanobacteria accumulate osmoprotectants like sucrose and glycine betaine to maintain cell integrity during dehydration^64^, while genome-encoded carbon and nitrogen fixation pathways contribute to nutrient cycling in dry soils^65^. Furthermore, interactions with plant growth-promoting rhizobacteria may strengthen root systems and enhance drought tolerance^66^. Collectively, genomic and functional evidence suggest that *Marileptolyngbya sp.* possesses adaptive features that enable it to stabilize soils, enhance nutrient availability, and promote plant resilience under drought.

To characterize the differentially accumulated strains under drought conditions, 47 endophytic bacterial strains were isolated and characterized from drought-treated cowpea roots. The 16 S rRNA gene sequence analysis revealed distinct bacterial compositions between the two culturing approaches used to isolate endophytic bacteria. The ACC deaminase-selective culturing method predominantly yielded strains belonging to Enterobacterales (Enterobacteriaceae), whereas the non-selective culturing approach yielded a more diverse bacterial community, primarily within the Gammaproteobacteria class. Most strains isolated on an ACC deaminase-selective medium clustered within a clade. This clustering suggests a potential genetic similarity among ACC deaminase-producing bacteria.

Functional characterization of the isolates revealed varying levels of osmotic stress tolerance and diverse plant growth-promoting activities. Several strains significantly improved wheat biomass in pot experiments, underscoring their potential as biostimulators under drought conditions. Targeted isolation of endophytic bacteria identified by metagenomic analysis enabled their cultivation and subsequent functional and biotechnological evaluation^67^. Consistently, *Enterobacter* spp., *Bacillus* spp., *Leclercia adecarboxylata*, and *Stenotrophomonas* spp. were detected as ASVs and successfully recovered using culture-based methods. Because a universal medium was used for most isolations, apart from the ACC deaminase-selective medium, the absence of cyanobacteria was expected. This inability to isolate cyanobacterial species represents a methodological limitation that future work could address by incorporating selective media.

The bacterial taxa identified in this study align with endophytic bacteria previously reported to possess plant-beneficial traits in cowpea and other plant species. For example, *Bacillus* spp., including *P. filamentosa* (formerly *Bacillus* sp.), *Stenotrophomonas* spp., and *P. aryabhattai* have been isolated from cowpea and shown to possess drought tolerance and plant growth-promoting functions^17,68^. Likewise, *Citrobacter freundii*, identified here as an endophytic bacterium, has been documented in wild desert cacti (*Euphorbia trigona* Mill)^69^, and its co-application with silicon has been shown to mitigate drought stress in tomatoes (Ullah et al., 2016). These comparisons reinforce the relevance of the isolates recovered in this study and support their potential use in improving plant performance under water-limited conditions.

*Enterobacter* spp, another genus identified in this study, is crucial to mitigating plant drought stress. Specifically, *Enterobacter bugandensis*, which we isolated, has been previously characterized for its drought tolerance. A strain of this bacterium, WRS7, has demonstrated the ability to induce systemic tolerance in wheat (*Triticum aestivum*) under drought conditions^70^. Additionally, *E. cloacae* with ACC deaminase activity has been shown to significantly promote maize growth, improve gas exchange attributes, and enhance nutrient uptake under drought stress when applied with timber waste biochar^71^. Further, *E. ludwigii* and *Bacillus megaterium* have been reported to enhance *Medicago sativa* growth under drought stress by increasing antioxidant activity, nutrient uptake, and biochemical resilience^72^. *E. ludwigii* strain GAK2 has also demonstrated the ability to solubilize silicon and phosphorus, produce organic acids, IAA, and gibberellins (GA1, GA3), and promote seed germination and rice growth^73^. Moreover, *E. ludwigii* b3, found in wild rice rhizospheres, helps mitigate drought stress in cultivated rice by enhancing root development, improving physiological and biochemical parameters, and reshaping the rhizosphere microbial communities^74^.

Another endophytic bacterium characterized in this study, *Pantoea agglomerans* ANP8, was previously identified as a salinity- and drought-resistant bacterium isolated from *Medicago sativa* root nodules, possessing genes encoding multiple IAA production pathways and phosphate solubilization enzymes^75^.

*Providencia* spp., also isolated in this study, aligns with findings showing that *P. rettgeri* enhances drought tolerance in barley (*Hordeum vulgare*) by improving growth parameters, water retention, chlorophyll content, photosynthetic efficiency, and stress resilience while reducing plant sensitivity indexes^76^. Moreover, *P. rettgeri* promotes salt stress resilience in *Arabidopsis thaliana* by increasing growth parameters, producing IAA and siderophores, and solubilizing phosphate under salt-stress conditions^77^. Similarly, *P. vermicola*, characterized in this study, is consistent with the findings on strain ME1, which demonstrated plant growth-promoting activities under PEG-induced osmotic stress and significantly improved the growth of *Triticum durum* under drought conditions^78^.

The identification of *Stenotrophomonas* spp. in our study as a growth-promoting bacterium is consistent with previous research on *S. maltophilia*, which, in combination with *Azospirillum brasilense*, enhances wheat tolerance to drought stress by improving relative water content, leaf area, chlorophyll (a and b), ascorbic acid levels, and root protein patterns while reducing electrolyte leakage, malondialdehyde, and hydrogen peroxide accumulation^79^. *Stenotrophomonas* spp. strains have been shown to tolerate PEG concentrations up to 50% while maintaining plant growth-promoting abilities, significantly enhancing wheat seedling growth, including length, fresh and dry weight, and water content^80^. Additionally, *S. pavanii* strain DJL-M3, when combined with biochar, stabilizes rhizosphere soil homeostasis and promotes rice growth under carbendazim stress (Feng et al., 2023). *S. pavanii* has also demonstrated multiple PGPB traits, exhibiting tolerance to drought, high temperature, alkalinity, and salinity. Inoculation with this bacterium significantly improved the characteristics of cluster bean (*Cyamopsis tetragonoloba*) seedlings, increasing dry weight, plant water potential, and relative leaf water content while reducing antioxidant enzyme and proline levels, resulting in increased yield^81^.

The validation of our findings through comparisons with prior studies indicates the potential application of these bacterial isolates in sustainable agriculture, particularly in enhancing crop tolerance to drought and salinity stress.

In conclusion, this study emphasizes the role of root endophytic bacterial communities in enhancing cowpea drought resilience. Drought adversely affected cowpea growth and significantly decreased bacterial diversity, triggering a community shift characterized by the enrichment of the Cyanobacteriota phylum, with *Marileptolyngbya sina* emerging as the only significantly enriched species. The reduction in microbial diversity suggests that drought selectively enriches specific taxa, which may enhance plant resilience through specialized functions—a potential drought resilience mechanism driven by endophytic bacteria in cowpeas. The identified endophytic isolates exhibited plant growth-promoting traits consistent with their demonstrated potential to improve drought tolerance. However, it is still necessary to test these new bacterial isolates in the field to deepen our understanding of plant drought resilience and advance their potential use in biofertilizer production, thereby contributing to sustainable agriculture.

## Supplementary Information

Below is the link to the electronic supplementary material.

Supplementary Material 1

Supplementary Material 2

Supplementary Material 3

## Data Availability

The metagenomic data produced in this report were submitted to GenBank as a sequence read archive (SRA) under the BioProject ID: PRJNA1230384, Submission ID: SUB15147529. The 16rRNA gene sequence data were submitted to GenBank under the SUB15147703 submission number (PV203386-PV203432).

## Declarations

### Plant materials

The cowpea (*Vigna unguiculata*) seed cultivar used in this study, originally produced in Canada, was purchased from a local market in 2024. The collection and use of plant material in this study complied with all applicable regional and national regulations, and no additional permissions were required.

### Voucher specimens

Bacterial strains isolated in this study will be available upon request to other researchers for non-commercial use. Laboratory specialists confirmed that identification and strain information are provided in the manuscript.
