# Supplementary material for: Metagenomic and functional insights into root endophytic bacteria associated with drought stress in cowpea
Source: Sci Rep. 2026 Mar 22;16:14519. doi: 10.1038/s41598-026-45459-4 (PMC13149983; doi:10.1038/s41598-026-45459-4)
Supplement: Supplementary file 3 — Supplementary Material 3 [file 41598_2026_45459_MOESM3_ESM.docx]

**Table S3.** Endophytic bacterial strains isolated from cowpea roots were identified through 16S rRNA gene sequencing. The obtained sequences were compared with sequences in GenBank to determine their closest relatives and the percentage identity with known bacterial species. The classification was based on 16S rRNA identity thresholds for taxonomic units. The classified strains were all deposited in GenBank, and their respective accession numbers are listed in the table.

| **Strain** | **Closest Species** | **Identity %** | **GenBank Accession** | **Strain classification** | **GenBank deposit number** |
| --- | --- | --- | --- | --- | --- |
| VU-E1 | *Enterobacter bugandensis* | 99% | NR_148649.1 | *Enterobacter bugandensis* | PV203386 |
| VU-E2 | *Citrobacter freundii* | 97% | NR_117752.1 | *Citrobacter* spp. | PV203419 |
| VU-E3 | *Providencia rettgeri* | 97% | NR_042413.1 | *Providencia* spp. | PV203387 |
| VU-E4 | *Enterobacter bugandensis* | 94% | NR_148649.1 | Enterobacteriaceae | PV203388 |
| VU-E5 | *Enterobacter bugandensis* | 98% | NR_148649.1 | *Enterobacter* spp. | PV203389 |
| VU-E6 | *Pantoea agglomerans* | 99% | NR_111998.1 | *Pantoea agglomerans* | PV203390 |
| VU-E7 | *Enterobacter bugandensis* | 94% | NR_148649.1 | Enterobacteriaceae | PV203391 |
| VU-E8 | *Providencia rettgeri* | 99% | NR_042413.1 | *Providencia* spp. | PV203392 |
| VU-E9 | *Enterobacter bugandensis* | 96% | NR_148649.1 | *Enterobacter* spp. | PV203420 |
| VU-E10 | *Enterobacter cloacae* | 95% | NR_118568.1 | *Enterobacter* spp. | PV203421 |
| VU-E11 | *Enterobacter bugandensis* | 99% | NR_148649.1 | *Enterobacter bugandensis* | PV203393 |
| VU-E12 | *Enterobacter quasihormaechei* | 99% | NR_180451.1 | *Enterobacter quasihormaechei* | PV203422 |
| VU-E13 | *Enterobacter ludwigii* | 97% | NR_042349.1 | *Enterobacter* spp. | PV203423 |
| VU-E14 | *Providencia vermicola* | 86% | NR_042415.1 | Gammaproteobacteria | PV203424 |
| VU-E15 | *Providencia rettgeri* | 93% | NR_115880.1 | Enterobacteriaceae | PV203394 |
| VU-E16 | *Enterobacter quasihormaechei* | 91% | NR_180451.1 | Enterobacteriaceae | PV203425 |
| VU-E17 | *Providencia rettgeri* | 100% | NR_042413.1 | *Providencia rettgeri* | PV203395 |
| VU-E18 | *Providencia rettgeri* | 98% | NR_115880.1 | *Providencia* spp. | PV203396 |
| VU-E19 | *Bacillus cabrialesii* | 98% | NR_180419.1 | *Bacillus* spp. | PV203397 |
| VU-E20 | *Providencia vermicola* | 91% | NR_042415.1 | Enterobacterales | PV203426 |
| VU-E21 | *Providencia rettgeri* | 100% | NR_042413.1 | *Providencia rettgeri* | PV203398 |
| VU-E22 | *Providencia rettgeri* | 100% | NR_042413.1 | *Providencia rettgeri* | PV203399 |
| VU-E23 | *Providencia rettgeri* | 99% | NR_042413.1 | *Providencia rettgeri* | PV203400 |
| VU-E24 | *Providencia vermicola* | 94% | NR_042415.1 | Enterobacteriaceae | PV203427 |
| VU-E25 | *Providencia rettgeri* | 97% | NR_115880.1 | *Providencia* spp. | PV203428 |
| VU-E26 | *Enterobacter bugandensis* | 100% | NR_148649.1 | *Enterobacter bugandensis* | PV203401 |
| VU-E27 | *Stenotrophomonas pavanii* | 86% | NR_118008.1 | Gammaproteobacteria | PV203429 |
| VU-E28 | *Providencia rettgeri* | 98% | NR_042413.1 | *Providencia* spp. | PV203402 |
| VU-E29 | *Enterobacter quasihormaechei* | 96% | NR_180451.1 | *Enterobacter* spp. | PV203403 |
| VU-E30 | *Providencia rettgeri* | 96% | NR_042413.1 | *Providencia* spp. | PV203404 |
| VU-E31 | *Providencia vermicola* | 92% | NR_042415.1 | Enterobacteriaceae | PV203430 |
| VU-E33 | *Ochrobactrum teleogrylli* | 98% | NR_174270.1 | *Ochrobactrum* spp. | PV203405 |
| VU-E34 | *Stenotrophomonas pavanii* | 99% | NR_118008.1 | *Stenotrophomonas pavanii* | PV203406 |
| VU-E35 | *Stenotrophomonas pavanii* | 98% | NR_118008.1 | *Stenotrophomonas* spp. | PV203407 |
| VU-E36 | *Leclercia adecarboxylata* | 85% | NR_104933.1 | Gammaproteobacteria | PV203432 |
| VU-E37 | *Enterobacter bugandensis* | 99% | NR_148649.1 | *Enterobacter* spp. | PV203408 |
| VU-E38 | *Providencia rettgeri* | 99% | NR_042413.1 | *Providencia rettgeri* | PV203409 |
| VU-E39 | *Providencia rettgeri* | 95% | NR_042413.1 | *Providencia* spp. | PV203410 |
| VU-E40 | *Stenotrophomonas pavanii* | 83% | NR_118008.1 | Proteobacteria | PV203411 |
| VU-E41 | *Stenotrophomonas pavanii* | 82% | NR_118008.1 | Proteobacteria | PV203431 |
| VU-E42 | *Providencia rettgeri* | 95% | NR_042413.1 | *Providencia* spp. | PV203412 |
| VU-E43 | *Enterobacter bugandensis* | 100% | NR_148649.1 | *Enterobacter bugandensis* | PV203413 |
| VU-E44 | *Enterobacter bugandensis* | 99% | NR_148649.1 | *Enterobacter bugandensis* | PV203414 |
| VU-E45 | *Enterobacter bugandensis* | 97% | NR_148649.1 | *Enterobacter* spp. | PV203415 |
| VU-E47 | *Providencia vermicola* | 91% | NR_042415.1 | Enterobacterales | PV203416 |
| VU-E48 | *Stenotrophomonas maltophilia* | 99% | NR_112030.1 | *Stenotrophomonas maltophilia* | PV203417 |
| VU-E49 | *Enterobacter bugandensis* | 100% | NR_148649.1 | *Enterobacter bugandensis* | PV203418 |
